# Prevention and control of multidrug-resistance tuberculosis in Ethiopia: Patients’ perspectives from the Oromia region

**DOI:** 10.1371/journal.pone.0322054

**Published:** 2025-05-12

**Authors:** Reta Angessa Beyene, Elsie Janse van Rensburg

**Affiliations:** Department of Health Studies, University of South Africa (UNISA), Pretoria, South Africa; Management Sciences for Health (MSH), ETHIOPIA

## Abstract

**Background:**

Multi-drug-resistant tuberculosis (MDR-TB) is one of the biggest challenges worldwide to end tuberculosis. It is vital to understand the challenges and opportunities of patients during MDR-TB treatment to enhance prevention and control efforts. The gap in research on the challenges and opportunities of patients during the screening, diagnosis, referral, and follow-up of MDR-TB prompted this study.

**Purpose:**

The purpose of the study was to assess the challenges and opportunities for patients with MDR-TB during the diagnosis and treatment of MDR-TB in the Oromia region of Ethiopia.

**Methods:**

A qualitative approach was applied. The data were collected from 30 MDR-TB patients from 1 to 30 April 2022 using semi-structured interviews after written informed consent was signed by each participant to understand the challenges and opportunities of MDR-TB treatment. Data was analysed by thematic analysis using ATLAS.ti software.

**Result:**

This article identifies challenges that include delays in diagnosis and treatment initiation due to inadequate diagnostic services, physical inaccessibility, and financial problems faced by patients to pay for transport, food, diagnosis, and accommodation. Other challenges included lack of psychosocial support, shortage of healthcare providers, poor communication, drug side effects, and interruption of food and housing support. In addition, participants mentioned opportunities, which include the availability of free diagnosis, treatment, and admission; availability of transport; food and housing allowance; and use of an ambulance for referral.

**Conclusion:**

This study filled a research gap in Ethiopia by identifying challenges and opportunities during the MDR-TB treatment program. The MDR-TB treatment program should focus on improving inadequate screening and resources, shortage of healthcare providers, delays in the referral process, and non-compliance of patients.

## 1. Introduction

An incredible 10 million individuals worldwide suffer from tuberculosis (TB) each year. Even though it is preventable and treatable, 1.5 million people die from tuberculosis each year; in 2022, it was the second most common infectious disease-related cause of death globally [1, 2, 3, 4, 5]. However, a genetic change caused some TB bacterial strains to become resistant to standard drugs [6].

TB is the biggest cause of death globally and the second leading cause from a single infectious agent, ranking higher than HIV/AIDS [1,5]. Globally, 7.5 million people received a new TB diagnosis in 2022, and 1.3 million of them passed away that year [5]. Rifampicin resistance is a serious concern, and drug-resistant TB remains a hazard to public health. Global estimates for the percentage of MDR-TB in 2022 were 3.3% in newly diagnosed patients and 17% in previously treated cases [5].

Despite having some of the highest rates of TB and TB/HIV, Ethiopia left the high MDR-TB burden countries in 2022 [5]. According to nationwide drug-resistant research, the prevalence of MDR-TB is 2.7% among newly diagnosed cases and 14.0% among patients who require retreatment [7,8].

With a treatment success rate of over 70% in 2021, higher than the global average of 58% in the same year, Ethiopia’s MDR-TB detection rate has significantly improved. However, compared to the anticipated number of cases, the number of MDR-TB cases that are discovered and begin treatment each year is incredibly low [3,5,9].

An effective prevention and control of MDR-TB must include early screening to detect drug resistance and efficient treatment of patients with MDR-TB [5,10]. In a study done in Vietnam, less than 20% of the projected total cases of MDR-TB cases were detected and treated with second-line treatment [10]. Factors that influence inadequate detection and treatment include inadequate TB screening capacity, inadequate communication, delay in treatment initiation, lack of adequately trained health workers, and lack of budget [5, 10, 11].

After intensive online and local searches for literature, the challenges and opportunities for patients with MDR-TB during diagnosis and treatment were never evaluated in the Ethiopian MDR-TB program. This study will fill the literature gap in terms of challenges and opportunities for MDR-TB patients.

## 2. Methods

### 2.1. Study area and period

The study was carried out in the Oromia region in Ethiopia, which had 23 MDR-TB treatment initiating centers (TICs). The Oromia region is the largest region with a population of 42,744,832, which is 39% of the country’s total population and divided into 21 administrative zones and 334 districts [12]. The study included 30 patients with MDR-TB who were treated in 15 TICS, and data were collected from 1 to 30 April 2022.

### 2.2. Study design and population

This was a qualitative study conducted with 30 patients with MDR-TB in 15 MDR-TB TICS in the Oromia region.

### 2.3. Sample size determination and sampling technique

Purposive sampling is one of the nonprobability sampling techniques that is frequently used to select the sample to obtain more relevant and useful data in a qualitative approach and in accordance with the study objectives [13,14]. Similarly, the Oromia region was purposively selected from 11 regions based on the largest population in the country. Two MDR-TB patients currently on treatment were purposively selected for interviews from each MDR-TB TICs. Data were collected from 30 patients with MDR-TB in 15 MDR-TB initiating centres (TICs) out of a total of 23 TICs in the Oromia region. The data were collected in April 2022. The inclusion criteria include MDR-TB patients who were on treatment in treatment centres in the Oromia region. The exclusion criteria included MDR-TB who had completed their treatment, critically sick during data collection, and age less than 18 years.

### 2.4. Data collection and analysis

Data was collected from two MDR-TB patients per TICs until data saturation, and two more interviews were conducted to ensure data saturation. Thematic analysis was applied in this study using ATLAS. ti software to code themes, subthemes, and categories with verbatim quotations. The researcher and co-coder generated initial codes, revised the codes, looked for similarities and patterns, and combined some of the codes/categories based on agreement. Then developed themes by grouping similar codes, further examined the adequacy of evidence for each developed theme and finally defined and renamed the final themes based on evidence available from the data. The analysis began during the data collection phase.

### 2.5. Ethical considerations

Ethical approval was obtained from the College Research Ethics Committee of the University of South Africa in 2022 (HSHDC/959/2020). A request was made to the Oromia Regional Health Bureau, and the necessary authority was granted to begin the research. Finally, the hospital and a health centre director were approached for permission and used as gatekeepers to interview MDR-TB patients. Interviews were conducted with each patient after receiving written informed consent. The participants were informed during informed consent that they might not directly benefit from the study result; however, the result of the study will benefit the public. During interviews, the right to fair treatment, anonymity, privacy, and confidentiality was ensured. To ensure privacy and confidentiality, data was kept on password-protected computers and under lock and key and will be kept for five years.

### 2.6. Rigor and trustworthiness

The reliability of the interview guide was enhanced through a pre-test to ensure clarity of the questions. To ensure dependability, the written and audio data were also kept by the researcher for further verification. Both the procedure for gathering field data and the steps involved in data processing were documented in detail. The responses of the participants were reflected in verbal quotes and included in the report as an extra precaution to ensure confidentiality. Transferability was demonstrated by providing a thorough explanation of the data collection methods and instruments utilized and the steps and processes used for the data analysis and presentation of the patients with MDR-TB.

## 3. Result

### 3.1. Demographic profile of the participants

A total of 30 MDR-TB patients (13 male and 17 female) were interviewed; among them, 22 out of 30 (73%) of the patients were in the age group 25–54 years. There were 5 participants aged 18–24 years, three participants 55 years and older, and there were no participants below age 18 years.

The interview resulted in two key themes; theme 1- challenges, and theme 2- opportunities.

### 3.2. Theme 1: MDR-TB treatment challenges

Challenges (Theme 1) were classified into three categories, as reflected in “Table 1”: (i) during diagnosis and treatment; (ii) referral process; and (iii) follow-up of treatment. The first category, namely challenges during diagnosis and treatment, had three subcategories: (i) delay in initiating MDR-TB treatment; (ii) lack of diagnostic services; and (iii) inadequate psychological support and limited communication.

**Table 1:** Major themes, categories, and subcategories for semi-structured interviews with patients with MDR-TB.

Themes	Categories	Subcategories
1.MDR-TB treatment challenges	1.1 Challenges during diagnosis and treatment	1.1.1 Delay in initiating MDR-TB treatment 1.2.2 Absence of diagnostic services
		1.1.3 Insufficient psychological support and limited communication
	1.2 Challenges during the referral process	1.2.1 Delay in timely patient and sample referral
		1.2.2 Physical and financial inaccessibility to treatment initiating centers (TIC)
	1.3 Challenges during treatment follow-up	1.3.1 Drug side effects
		1.3.2 Interruption of food and housing support
2.MDR-TB treatment opportunities	2.1 Opportunities for the initiation and adherence to treatment	2.1.1 Availability of free diagnostic, treatment, and admission
		2.1.2 Availability of transport and housing allowance
	2.2 Conducive environments during referrals	2.2.1 Use of ambulance for referral and being escorted
	2.3 Conducive environments during treatment follow-up	2.4.1 Food and nutritional support

#### 3.2.1. Challenges during diagnosis and treatment.

This category included three subcategories.

3.2.1.1. ***Delay in initiating MDR-TB treatment***. The delay in initiation of MDR-TB treatment as explained by the participants means the time gap between initial symptoms of TB and diagnosis and the start of MDR-TB medication.

As revealed by MDR-TB patients, the treatment delay was because of patients’ initial visits to traditional (herbal) treatments, delay in diagnosis, financial problems for transport and physical distance of treatment centers, and inadequate knowledge of private healthcare providers.

*‘... my health condition did not improve, and I went back to the health facility, and finally I was diagnosed with TB after one year and five months.’ #P12*

‘... *I couldn’t get treatment for my problem [MDR-TB] for many months because the health workers did not identify my problem.’ #15*

3.2.1.2. ***Absence of diagnostic services.*** The absence of diagnostic facilities was one of the challenges mentioned by the participants. Advanced diagnosis, including X-rays, heart imaging, drug susceptibility tests, and GeneXpert, were absent in the lower health facilities. Other diagnostic services, such as blood and sputum tests, were interrupted due to the shortage of reagents.

*‘... I was diagnosed after seven months.’ #P17*

‘… I couldn’t get diagnostic facilities at the health center… I have to travel more than 100 kilometers by public transport to get the service in a hospital.’ *#P25*

3.2.1.3. ***Inadequate psychological support.*** Inadequate psychological support was one of the challenges mentioned by patients with MDR-TB during MDR-TB treatment. Participants highlighted inadequate counseling about the duration of treatment, identification and management of drug side effects, and loneliness in isolation centers.

*‘...it was one of the most stressful times in my life... I received very little psychological support and counseling from health care providers.’* #P7

#### 3.2.2. Challenges during the referral process.

Two subcategories were discussed under this category.

3.2.2.1. ***Delay in timely patient and sample referral***. As mentioned by MDR-TB patients, these delays were caused by late or incorrect diagnosis, causing delayed decision-making and referrals. The absence of an ambulance, the absence of a referral protocol, and poor communication between health facilities were also among the reasons for delayed referral.

*‘... it took me four months to get the hospital’s test result... I think my result [the MDR-TB test result] took so long due to the absence of good communication between them [the health facilities]. ‘#P11*

3.2.2.2. ***Physical and financial inaccessibility of MDR-TB treatment centers***. The distance from MDR-TB treatment centers, the lack of money for transport, and food and security issues due to armed conflict were mentioned by participants as challenges for timely referral.

*‘…I couldn’t get money for transport as I was sick in bed… it took me more than a month before I got money from a relative. When I arrived at the health facility, I was admitted and finally diagnosed with this problem [MDR-TB].’* #P11

*‘... I couldn’t get diagnostic facilities in the health center... I have to travel more than 100 km by public transport to get the service in a hospital. ‘#P25*

*‘…I had to discontinued treatment for one month because there was no transport due to conflicts between armed groups and the government…Finally my doctor sent me the medicines through a member of armed group.” #8*

#### 3.2.3. Challenges during treatment follow-up.

Two subcategories were discussed under this category.

3.2.3.1. ***Identification and management of side effects.*** Drug side effects such as gastric pain, skin rash, joint pain, and hearing loss were among the most frequently mentioned challenges for the adherence of MDR-TB medications by patients with MDR-TB. Many of the participants stopped MDR-TB treatment due to drug side effects, as they were not informed of it and did not know how to manage it.

*‘…after I started taking the drugs [MDR-TB drugs], I developed severe gastric pain and vomiting, because of that I interrupted taking my treatment.’ #*17

*‘…I have developed severe pain on my left leg after I started taking the treatment and I couldn’t walk at all.’ #29*

3.2.3.2. ***Interruption of food, transport, and housing support***. The interruption of financial support by treatment centers to patients with MDR-TB for food and housing was one of the challenges faced by patients with MDR-TB during follow-up of treatment, as patients could not afford food, transport, and accommodation without allowances.

*‘…there was a time the health facility stopped providing us housing allowance, and at that time I had to walk more than 10 kilometers for and back to my house to take the treatment [MDR-TB treatment].’ #25*

### 3.3. Theme 2: MDR-TB treatment opportunities

The opportunities (Theme 2) were classified into three categories as displayed in “Table 1”: (i) opportunities for treatment initiation and adherence; (ii) conducive environments during referrals; and (iii) conducive environments during treatment follow up.

#### 3.3.1. Opportunities for treatment initiation and adherence.

This category included two subcategories.

3.3.1.1. ***Availability of free diagnostic, treatment, and admission***. Participants mentioned the availability of free MDR-TB diagnostic and treatment services was one of the opportunities for them to initiate and adhere to treatment.

*‘…all the treatments were free of charge, including treatments for drug side effects; it was a good opportunity for me to start treatment and continue with the treatment.*’ #P8

3.3.1.2. ***Availability of transport and housing allowance***. Availability of transport and housing allowance was one of the motivating factors mentioned by MDR-TB patients to initiate treatment and adherence to treatment follow ups.

*‘…I was provided with money for transport from and to my house… It was not enough; however, I couldn’t afford the transport cost out of my pocket and should have discontinued treatment.’* #P19

#### 3.3.2. Conducive environments during referral.

One subcategory was discussed under this category.

3.3.2.1. ***Use of ambulance for referral and being escorted***. Participants indicated that transport arrangements and being escorted by health workers to and from hospitals were motivational factors during referral.

*‘…I was referred to the hospital… with an ambulance, and a health worker also escorted me… I got a good reception at the hospital.’* P17

#### 3.3.3. Conducive environments during treatment follow-up.

One subcategory was discussed under this category.

3.3.3.1. ***Food and nutritional support***. Participants mentioned that they were provided with basic food items on a monthly basis, which was an opportunity for them not to stop their follow-up due to a lack of food.

*‘…The hospital provided me food support monthly during my treatment follow-up. The hospital gave me food items support like edible oil, sugar, pasta, flour, and rice during follow-up.’* #P23

## 4. Discussion

The result was divided into two themes: MDR-TB challenges and opportunities during MDR-TB treatment. The challenges include delays in initiating treatment, lack of diagnostic services, inadequate psychological support, shortage of healthcare providers, and delays in referral processes. The physical and financial inaccessibility of treatment centers was also identified as a challenge for MDR-TB treatment. The absence of advanced diagnostic facilities, such as X-rays, heart imaging, drug susceptibility tests, and GeneXpert, was the major challenges during diagnosis. Inadequate management of drug side effects, which led to interruption of treatment, was among the challenges for treatment follow-up. The opportunities for treatment initiation and continuation include the availability of free diagnostic, treatment, and admission services, as well as the availability of food, transport, and housing allowances. Overall, the study highlights the need for improved psychological and financial support for MDR-TB patients to ensure successful treatment outcomes.

In this study, one of the challenges during MDR-TB treatment was the delays in initiation of MDR-TB treatment because of low community awareness about TB/MDR-TB, patients’ initial visits to traditional treatment as a result, delay in diagnosis, delay in laboratory results, physical and financial inaccessibility of MDR-TB treatment initiating centers, and inadequate knowledge of healthcare providers. A participant mentioned a delay of more than one year before being diagnosed and treated for MDR-TB. Similarly, studies conducted in China showed an average treatment delay of up to 33 days before starting MDR-TB treatment [15].

The studies showed there was a gap in knowledge among communities, which leads to delayed diagnosis and treatment, worsening the patients’ condition [15]. Hence, increasing community outreach programs focused on TB education and engaging traditional healers in the health system to educate patients on the importance of timely diagnosis and treatment are vital parts of prevention and control.

Similar to this study, which showed treatment delays were associated with patients’ financial problems for transport and accommodation, low community awareness of TB, delays in diagnostic results, and inadequate knowledge of health workers on MDR-TB treatment, studies conducted in Ethiopia and China showed the treatment delays were linked to patients low awareness, poverty, financial problems, delays in diagnostic results, and lack of knowledge of healthcare providers [15, 16, 17]. Other studies conducted in Indonesia and Mozambique showed distance from health facilities and transport costs were associated with delays in treatment initiation, which is similar to this study, which found MDR-TB diagnosis and treatment were delayed due to the inaccessibility of treatment centers [18].

Economic hardship for transport and accommodation closed off access to necessary MDR-TB treatment, perpetuating the cycle of illness. Advocating for financial assistance programs for low-income patients, including transportation vouchers or subsidies, can alleviate the burden on patients.

Furthermore, like this study, which showed patients initially visited traditional (herbal) therapies, which delayed their diagnosis and treatment for as long as a year, studies conducted in South Africa and Nigeria showed that due to low awareness, MDR-TB patients initially visit traditional or spiritual treatments. As a result, they visit MDR-TB treatment centers very late [19, 20, 21, 22].

Collaborate with traditional healers to integrate their practices with modern healthcare and educate them about TB and the risks of delays in treatment, encouraging referrals to health facilities when symptoms are present to improve the delays due to initial treatment with traditional (herbal) therapies.

Delay in diagnosis of MDR-TB patients because of absence of diagnostic services, delay in referral of patient and sample, delay in decision-making, financial problems, absence of ambulance, and referral protocol was one of the findings in this study. Likewise, a study conducted in Ethiopia showed the absence of GeneXpert at the health center level was one of the reasons for the delay in the diagnosis of MDR-TB patients [16]. In this study, participants have mentioned a delay in diagnosis for seven months. Likewise, studies conducted in India, Tanzania, and Ghana showed that there was a lot of delay in sample referral and turnaround time (TAT) range from 38 to 51 days. Like this study, which showed the absence of referral protocol for delay in referral of a sample, the referral delays were related to the absence of a referral protocol in the studies conducted in India, Tanzania, Ghana, and Ethiopia [23, 24, 25].

Lack of advanced diagnostic facilities hinders the timely diagnosis of MDR-TB, contributing to treatment delays. Investing in diagnostic infrastructure like GeneXpert at healthcare centers to improve accessibility and speed up the diagnostic process is crucial to improving TB control.

In this study, availability of free diagnostic and treatment services was mentioned as a motivational factor to start MDR-TB treatment and adhere to treatment follow-up. Similarly, a study done in Nigeria showed the availability of a free MDR-TB isolation center as a motivational factor for initiating MDR-TB treatment [26].

Free diagnostic and treatment services were seen by patients as vital incentives for to seek care and promoting these services widely to ensure that the community is aware and can take advantage of them is paramount for MDR-TB prevention and control.

This study also found that housing and transport allowances provided to patients during MDR-TB treatment follow-up were motivational factors for treatment adherence. Similarly, studies conducted in Ethiopia and India showed that transport allowance was a motivational factor for enhanced treatment success [10,27]. Other studies conducted in Bangladesh and Pakistan also showed that food and transport allowances during MDR-TB treatment follow-up as motivational factors for treatment adherence [28,29].

Another motivational factor for treatment adherence in this study was food and nutritional support. Many participants have mentioned that without food and nutritional support from the treatment centres, they should have discontinued their treatment. Similarly, studies done in India, Bangladesh, and Pakistan showed food support for MDR-TB patients was among the motivational factors to adhere to treatment follow-ups [27, 28, 29].

Furthermore, the presence of an ambulance and being escorted by healthcare providers was identified as one of the motivational factors during referral. Similarly, according to a study done in Ethiopia, the presence of an ambulance and being escorted by a healthcare professional were among the motivational factors for MDR-TB patients [5].

Having support during the referral process increased the likelihood of patients attending necessary appointments, so it is important to establish protocols for regular follow-ups post-referral to reinforce patient engagement and adherence.

## 5. Limitations

One of the limitations of the study was that it did not include under-18 years old participants for the qualitative study, which might have missed the challenges during MDR-TB treatment in the under-18 years age group, which might be worse than for those above 18 years. The other limitation is that because of the nature of the study, there might be a self-report bias of qualitative study participants due to memory failure, a social desirability bias, or inaccurate reporting.

## 6. Conclusion

The study highlights the challenges faced by MDR-TB patients, including delays in treatment, lack of diagnostic services, inadequate psychological support, shortage of healthcare providers, and referral delays. It also emphasizes the need for improved psychological and financial support to ensure successful treatment outcomes. Expanding insurance coverage would reduce financial barriers, while decentralizing diagnosis and treatment could make care more accessible, especially in rural areas. Adding psychologists to the healthcare system is crucial because DR-TB patients often face mental health struggles due to prolonged treatment. Opportunities for treatment initiation include free services and allowances.

This study filled a research gap in Ethiopia by identifying challenges and opportunities during MDR-TB treatment from the perspectives of the MDR-TB patients.

Financial support for food, transport, and accommodation helped to alleviate the burdens associated with treatment. Continuing and expanding these support programs, ensuring they are accessible to all patients in need will encourage adherence to treatment.
